# [Corrigendum] Propranolol induces hemangioma endothelial cell apoptosis via a p53-BAX mediated pathway

**DOI:** 10.3892/mmr.2025.13647

**Published:** 2025-08-06

**Authors:** Tian-Hua Yao, Parekejiang Pataer, Krishna Prasad Regmi, Xi-Wen Gu, Quan-Yan Li, Jing-Ting Du, Su-Meng Ge, Jun-Bo Tu

Mol Med Rep 18: 684–694, 2018; DOI: 10.3892/mmr.2018.9013

Following the publication of the above article, the authors have contacted the Editorial Office to explain that Fig. 4A was inadvertently included in this paper, where it had accurately appeared in a paper by the same research group published in 2012 in *Journal of Biochemical and Molecular Toxicology*. Secondly, the same fluorescence microscopy data had been included in Fig. 6B and C (these data were correctly shown in the figure for panel 6C).

The revised versions of Figs. 4 and 6, now featuring the correct data for Figs. 4A and 6B, are shown on the next page. Note that the correction of these data does not grossly affect either the results or conclusions reported in this study. All the authors agree with the publication of this corrigendum, and are grateful to the Editor of *Molecular Medicine Reports* for allowing them the opportunity to publish this. The authors regret that these errors were included in the paper, and apologize to the readership for any inconvenience caused.

## Figures and Tables

**Figure 4.**
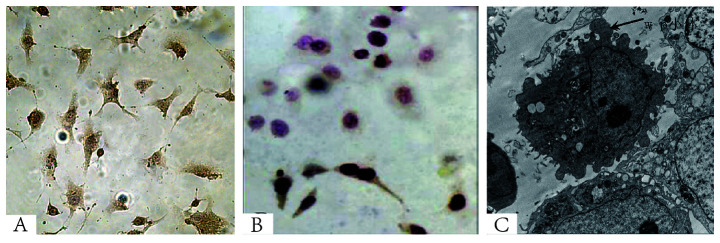
Immunocytochemical staining. (A) Yellow particles in immunocytochemical staining image indicate the presence of clotting factor VIII (magnification, ×400). (B) Negative control fibroblasts (magnification, ×400). (C) Weibel-Palade bodies (scanning electron microscopy; magnification, ×8,000). Weibel-Palade bodies are the storage granules of endothelial cells, the cells that form the inner lining of the blood vessels and heart. Therefore, Weibel-Palade bodies were considered to identify endothelial cells.

**Figure 6.**
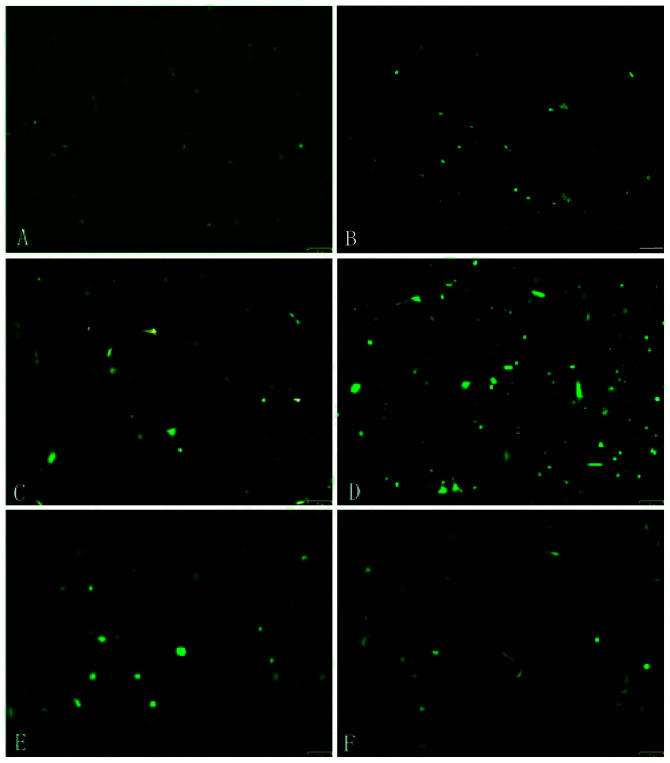
Fluorescent protein expression investigated using fluorescence microscopy. Picture A to F showed the fluorescent protein expression in (A) 6 h, (B) 12 h, (C) 18 h, (D) 24 h, (E) 32 h and (F) 40 h time intervals following transfection. The expression of fluorescence exhibited in (D) represents the greatest transfection ratio.

